# Supplementary material for: Enriched Red Wine: Phenolic Profile, Sensory Evaluation and In Vitro Bioaccessibility of Phenolic Compounds
Source: Foods. 2023 Mar 11;12(6):1194. doi: 10.3390/foods12061194 (PMC10048746; doi:10.3390/foods12061194)
Supplement: Supplementary file 1 [file foods-12-01194-s001.zip › Table S1. LOD and LOQ.docx]

**Table S1**. Linearity information of calibration curves.

| **Compound** | **m** | **b** | **r** | **L.O.D. (mg/mL)** | **L.O.Q. (mg/mL)** |
| --- | --- | --- | --- | --- | --- |
| Hydroxybenzoic acids |  |  |  |  |  |
| Gallic acid | 100000000 | -127541.0 | 0.9939 | 0.0017 | 0.0025 |
| Protocatechuic acid | 70000000 | -57925.0 | 0.9994 | 0.0014 | 0.0026 |
| Syringic acid | 9000000 | 3625.2 | 0.9990 | 0.0038 | 0.0135 |
| Ellagic acid | 30000000 | -69214.0 | 0.9987 | 0.0036 | 0.0065 |
| Hydroxycinnamic acids |  |  |  |  |  |
| Caffeic acid | 100000000 | -6722.4 | 0.9982 | 0.0004 | 0.0013 |
| Chlorogenic acid | 60000000 | -22815.0 | 0.9998 | 0.0010 | 0.0025 |
| Stilbenes |  |  |  |  |  |
| Resveratrol | 200000000 | -827829.0 | 0.9865 | 0.0043 | 0.0048 |
| Flavones |  |  |  |  |  |
| Luteolin | 200000000 | -2000000.0 | 0.9933 | 0.0102 | 0.0106 |
| Flavanones |  |  |  |  |  |
| Hesperetin | 300000000 | -641511.0 | 0.9973 | 0.0023 | 0.0026 |
| Naringenin | 2000000 | -1808.4 | 0.9997 | 0.0197 | 0.0635 |
| Flavanols |  |  |  |  |  |
| Myricetin | 100000000 | -1000000.0 | 0.9969 | 0.0104 | 0.0113 |
| Quercetin | 90000000 | -1000000.0 | 0.9925 | 0.0115 | 0.0125 |
| Rutin | 200000000 | -190659.0 | 0.9980 | 0.0011 | 0.0016 |
| Flavan-3-ols |  |  |  |  |  |
| Catechin | 100000000 | -125463.0 | 0.9983 | 0.0016 | 0.0025 |
| Epicatechin | 200000000 | -104706.0 | 0.9992 | 0.0007 | 0.0011 |
| Epicatechin gallate | 90000000 | -84240.0 | 0.9733 | 0.0014 | 0.0023 |
| Gallocatechin | 70000000 | -5420.9 | 0.9963 | 0.0006 | 0.0019 |
| Epigallocatechin | 100000000 | -16929.0 | 0.9993 | 0.0005 | 0.0014 |
| Procyanidin B1 | 100000000 | -74992.0 | 0.9990 | 0.0011 | 0.0020 |
| Procyanidin B2 | 100000000 | -61835.0 | 0.9984 | 0.0010 | 0.0019 |
| Procyanidin C1 | 70000000 | -46555.0 | 0.9987 | 0.0012 | 0.0025 |

m= slope; b= intercept; L.O.D.= limit of detection; L.O.Q.= Limit of quantification. L.O.D and L.O.Q. were calculated with the signal to noise ratio (S/N).
