# Supplementary material for: Enriched Red Wine: Phenolic Profile, Sensory Evaluation and In Vitro Bioaccessibility of Phenolic Compounds
Source: Foods. 2023 Mar 11;12(6):1194. doi: 10.3390/foods12061194 (PMC10048746; doi:10.3390/foods12061194)
Supplement: Supplementary file 1 [file foods-12-01194-s001.zip › Table S2. Sensory analysis.docx]

**Table S2**. Attributes and descriptors evaluated in wine samples.

| Attribute/  Descriptor | Definition | Standards |
| --- | --- | --- |
| Sight |  |  |
| Color | Red wine color is related to anthocyanin content in red wines. | Water, Rosé wine, and Cabernet Sauvignon wine |
| Brightness | Bright is defined as light dispersion into wine. | Water, Rosé wine, and Cabernet Sauvignon wine |
| Taste |  |  |
| Sweet | Glucose and fructose are the main responsible for sweetness in red wines. Sweetness can be intensified by ethanol. | Water, glucose solution at 8 and 16% |
| Bitter | Bitter taste in wines is affected by flavonoids content | Water, caffeine solution at 0.1 and 0.3% |
| Sourness | Organic acids such as tartaric, malic, lactic, and phenolic acids are the main responsible for sour taste in wines | Water, citric acid solution at 0.04 and 0.25% |
| Salty | Tartrates can dissociate into potassium, sodium and calcium and are involved in salty taste of wines | Water, sodium chloride solution at 0.4 and 0.75% |
| Mouthfeel |  |  |
| Astringency | Astringency is defined as a dryness or roughness sensation. Condensed tannins are involved in astringency by salivary proteins precipitation | Water, tannic acid solution at 0.1 and 0.35% |
| Body | Wine body is produced by ethanol and sugar content. Mannoproteins and polysaccharides from yest can affect the body | Water, non-saturated corn starch solution and supersaturated corn starch |
| Odor |  |  |
| Odor intensity |  |  |
| Alcohol | Ethanol odor | Water, ethanol solution at 50 and 96% |
| Prune | Prune odor | Cotton, crushed fresh plums, prunes |
| Wood | Wood odor | Cotton, 1g wood and 1g wet wood |
| Caramel | Caramel odor | Cotton, 1g caramel candy and 1g caramel popcorn |
| Fermented | Fermented odor | Water, fermented pineapple |
| Fruit | Fruit odor | Cotton, 1g fresh fruits, 1g dried fruit |
