# Supplementary material for: Enriched Red Wine: Phenolic Profile, Sensory Evaluation and In Vitro Bioaccessibility of Phenolic Compounds
Source: Foods. 2023 Mar 11;12(6):1194. doi: 10.3390/foods12061194 (PMC10048746; doi:10.3390/foods12061194)
Supplement: Supplementary file 1 [file foods-12-01194-s001.zip › Table S3. Spectral information.docx]

**Table S3.** Spectral information of compounds found in wine samples.

| **Compound** | **R.T. (min)** | **Formula** | **Precursor [M-H]** | **Experimental Mass** | **Theorical Mass** | **Score** | **Difference (ppm)** | **Fragments** |
| --- | --- | --- | --- | --- | --- | --- | --- | --- |
| Hydroxybenzoic acids |  |  |  |  |  |  |  |  |
| Gallic acid | 0.451 | C_7_ H_6_ O_5_ | 169.0149 | 170.0221 | 170.0215 | 95.93 | 3.51 | 169.0141; **125.0204** |
| 3-methyl-gallic acid | 1.058 | C_8_ H_8_ O_5_ | 183.0296 | 184.0370 | 184.0372 | 84.30 | -0.83 | 183.0229; **165.0184**, 139.0389 |
| 4-methyl-gallic acid | 4.024 | C_8_ H_8_ O_5_ | 183.0300 | 184.0374 | 184.0372 | 81.49 | 1.35 | 183.0292; **165.0184**; **139.0389** |
| Ethyl-gallate | 2.710 | C_9_ H_10_ O_5_ | 197.0447 | 198.0520 | 198.0528 | 96.53 | -4.25 | 197.0491; **169.0097** |
| Octyl-gallate | 1.632 | C_15_ H_22_ O_5_ | 281.1393 | 282.1463 | 282.1467 | 96.07 | -1.48 | **281.1397**; 229.0974; |
| Pyrogallol | 0.452 | C_6_ H_6_ O_3_ | 125.0245 | 126.0319 | 126.0317 | 98.04 | 1.72 | **125.0242**; |
| Gentisic acid | 2.540 | C_7_ H_6_ O_4_ | 153.0198 | 154.0271 | 154.0266 | 86.01 | 2.93 | **153.0192**; |
| *m*-hydroxybenzoic acid | 0.991 | C_7_ H_6_ O_3_ | 137.0240 | 138.0312 | 138.0317 | 79.93 | -3.72 | **137.0243**; |
| *p*-hydroxybenzoic acid | 1.664 | C_7_ H_6_ O_3_ | 137.0244 | 138.0316 | 138.0317 | 72.11 | -0.59 | **137.0243**; |
| Protocatechuic acid | 0.557 | C_7_ H_6_ O_4_ | 153.0195 | 154.0268 | 154.0266 | 92.00 | 0.98 | 153.0167; **109.0270** |
| Syringic acid | 1.400 | C_9_ H_10_ O_5_ | 197.0452 | 198.0524 | 198.0528 | 98.93 | -1.94 | **197.0511**; 120.0581 |
| Ellagic acid | 3.556 | C_14_ H_6_ O_8_ | 300.9989 | 302.0062 | 302.0063 | 97.14 | -0.23 | **300.9988**; 204.0659 |
| Vanillic acid | 3.754 | C_8_ H_8_ O_4_ | 167.0349 | 168.0420 | 168.0423 | 71.76 | -1.60 | **167.0356**; |
| Vanillic acid glucoside | 2.272 | C_14_ H_18_ O_9_ | 329.0877 | 330.0947 | 330.0951 | 92.45 | -1.18 | **329.0886**; 121.0297 |
| Homovanillic acid | 1.159 | C_9_ H_10_ O_4_ | 181.0499 | 182.0572 | 182.0579 | 84.51 | -3.80 | **181.0489**; 135.0443 |
| Hydroxycinnamic acids |  |  |  |  |  |  |  |  |
| Caffeic acid | 1.361 | C_9_ H_8_ O_4_ | 179.0349 | 180.0424 | 180.0423 | 93.86 | 0.68 | 179.0348; **135.0448**; 119.0363 |
| Caffeoyl-malate | 1.022 | C_13_ H_12_ O_8_ | 295.0463 | 296.0532 | 296.0532 | 87.44 | -0.03 | 295.0430; **164.0401**; 119.0363 |
| Methyl-ferulate | 5.405 | C_11_ H_12_ O_4_ | 207.0664 | 208.0737 | 208.0736 | 99.85 | 0.44 | **207.0633**; 161.0251; 119.0363 |
| *m*-coumaric acid | 6.181 | C_9_ H_8_ O_3_ | 163.0398 | 164.0469 | 164.0473 | 81.91 | -2.38 | **163.0450**; 119.0492 |
| **Compound** | **R.T. (min)** | **Formula** | **Precursor [M-H]** | **Experimental Mass** | **Theorical Mass** | **Score** | **Difference (ppm)** | **Fragments** |
| *p*-coumaric acid | 0.962 | C_9_ H_8_ O_3_ | 163.0396 | 164.0468 | 164.0473 | 80.19 | -3.08 | 163.0451; 119.0542 |
| Stilbenes |  |  |  |  |  |  |  |  |
| Resveratrol | 5.439 | C_14_ H_12_ O_3_ | 227.0712 | 228.0785 | 228.0786 | 99.73 | -0.63 | **227.0711**; 207.0664 |
| Dihydroresveratrol | 4.900 | C_14_ H_14_ O_3_ | 229.0864 | 230.0940 | 230.0943 | 94.64 | -1.16 | **229.0866**; 121.0300 |
| Resveratrol-glucoside | 4.425 | C_20_ H_22_ O_8_ | 389.1243 | 390.1314 | 390.1315 | 82.38 | -0.09 | **389.1243**; |
| Piceatannol | 3.383 | C_14_ H_12_ O_4_ | 243.0669 | 244.0738 | 244.0736 | 70.14 | 1.17 | **243.0662**; |
| Astringin | 3.754 | C_20_ H_22_ O_9_ | 405.1206 | 406.1280 | 406.1264 | 75.31 | 3.89 | **405.1732**; |
| ε-Viniferin | 4.597 | C_28_ H_22_ O_6_ | 453.1339 | 454.1418 | 454.1416 | 88.64 | 0.41 | **453.1334**; **435.1303**;317.0299 |
| δ-Viniferin | 5.304 | C_28_ H_22_ O_6_ | 453.1335 | 454.1406 | 454.1416 | 81.61 | -2.36 | **453.1222**; 331.0463 |
| Resveratrol dimer | 5.844 | C_28_ H_22_ O_6_ | 453.1342 | 454.1411 | 454.1416 | 76.79 | -1.25 | **453.1340**; 357.1328 |
| Phenylethanoids |  |  |  |  |  |  |  |  |
| Tyrosol | 4.560 | C_8_ H_10_ O_2_ | 137.0606 | 138.0678 | 138.0681 | 98.70 | -2.21 | **137.0608**; |
| Hydroxytyrosol | 0.586 | C_8_ H_10_ O_3_ | 153.0551 | 154.0623 | 154.063 | 93.65 | -4.62 | **153.0345**; |
| Coumarins |  |  |  |  |  |  |  |  |
| Esculetin | 1.359 | C_9_ H_6_ O_4_ | 177.0198 | 178.0270 | 178.0266 | 86.36 | 2.32 | **177.0191**; |
| Esculetin-glucoside | 4.054 | C_15_ H_16_ O_9_ | 339.0721 | 340.0789 | 340.0794 | 74.81 | -1.61 | **339.0716**; |
| Flavones |  |  |  |  |  |  |  |  |
| Luteolin | 5.271 | C_15_ H_10_ O_6_ | 285.0419 | 286.0487 | 286.0477 | 90.13 | 3.45 | **285.0400**; 271.0599 |
| Equol | 6.416 | C_15_ H_14_ O_3_ | 241.0868 | 242.0941 | 242.0943 | 85.55 | -0.90 | **241.0868**; |
| Tetrahydroxyisoflavone | 4.428 | C_15_ H_12_ O_6_ | 287.0562 | 288.0637 | 288.0634 | 82.81 | 1.06 | **287.0559**; |
| Pentahydroxyisoflavone | 3.788 | C_15_ H_10_ O_7_ | 301.0349 | 302.0424 | 302.0426 | 81.86 | -0.87 | **301.0355**; |
| Flavanones |  |  |  |  |  |  |  |  |
| Erodictyol | 1.602 | C_15_ H_12_ O_6_ | 287.0564 | 288.0635 | 288.0634 | 81.10 | 0.21 | **287.0533**; |
| Erodictyol-7-glucoside | 2.040 | C_21_ H_22_ O_11_ | 449.1079 | 450.1150 | 450.1162 | 88.40 | -2.58 | **449.1081**; |
| Homoerodictyol | 4.158 | C_16_ H_14_ O_6_ | 301.0715 | 302.0787 | 302.0790 | 84.05 | -1.27 | **301.0723**; 241.1084 |
| **Compound** | **R.T. (min)** | **Formula** | **Precursor [M-H]** | **Experimental Mass** | **Theorical Mass** | **Score** | **Difference (ppm)** | **Fragments** |
| Hesperetin | 4.129 | C_16_ H_14_ O_6_ | 301.0714 | 302.0786 | 302.0790 | 84.20 | -1.35 | **301.0713**; |
| Naringenin | 5.814 | C_15_ H_12_ O_5_ | 271.0608 | 272.0682 | 272.0685 | 85.17 | -1.05 | 271.0615; **215.1286** |
| Naringenin-glucoside | 4.664 | C_21_ H_22_ O_10_ | 433.1139 | 434.1210 | 434.1213 | 70.51 | -0.66 | **433.1140**; |
| Naringin | 3.860 | C_27_ H_32_ O_14_ | 579.1703 | 580.1771 | 580.1792 | 87.14 | -3.67 | **579.1706**; 551.1414 |
| Flavonols |  |  |  |  |  |  |  |  |
| Astilbin | 3.889 | C_21_ H_22_ O_11_ | 449.1085 | 450.1157 | 450.1162 | 96.02 | -1.05 | **449.1079**; |
| Isorhamnetin | 6.012 | C_16_ H_12_ O_7_ | 315.0509 | 316.0582 | 316.0583 | 98.87 | -0.37 | 315.0508; **285.0398**; **239.0922;** |
| Isorhamnetin-glucoside | 4.225 | C_22_ H_22_ O_12_ | 477.1036 | 478.1110 | 478.1111 | 98.63 | -0.33 | **477.1031**; 450.1061 |
| Kaempferol | 5.911 | C_15_ H_10_ O_6_ | 285.0403 | 286.0478 | 286.0477 | 98.75 | 0.06 | **285.0400**; 215.1286 |
| Kaempferol-3-glucoside | 4.192 | C_21_ H_20_ O_11_ | 447.0932 | 448.1004 | 448.1006 | 81.57 | -0.32 | **447.0934**; 439.0681 |
| Kaempferol-7-glucuronide | 4.192 | C_21_ H_18_ O_12_ | 461.0733 | 462.0806 | 462.0798 | 77.04 | 1.69 | **461.0725**; |
| Laricitrin | 5.303 | C_16_ H_12_ O_8_ | 331.0458 | 332.0531 | 332.0532 | 98.85 | -0.30 | **331.0455**; |
| Laricitrin-3-glucoside | 3.855 | C_22_ H_22_ O_13_ | 493.0981 | 494.1055 | 494.1060 | 98.10 | -1.06 | **492.0995**; 477.0688 |
| Myricetin | 4.466 | C_15_ H_10_ O_8_ | 317.0303 | 318.0376 | 318.0376 | 99.72 | 0.22 | **317.0307**; 137.0609 |
| Quercetin | 5.304 | C_15_ H_10_ O_7_ | 301.0357 | 302.0429 | 302.0426 | 99.28 | 0.75 | **301.0353**; 299.0198 |
| Quercetin-3-galactoside | 3.822 | C_21_ H_20_ O_12_ | 463.0880 | 464.0952 | 464.0955 | 98.60 | -0.62 | **463.0873**; 441.0820 |
| Quercetin-3-glucuronide | 3.759 | C_21_ H_18_ O_13_ | 477.0672 | 478.0744 | 478.0747 | 98.81 | -0.69 | **477.0666**; 463.0873 |
| Syringetin | 5.911 | C_17_ H_14_ O_8_ | 345.0611 | 346.0687 | 346.0689 | 94.06 | -0.35 | **345.0609**; 327.2169 |
| Syringetin-3-glucoside | 4.231 | C_23_ H_24_ O_13_ | 507.1143 | 508.1214 | 508.1217 | 98.81 | -0.64 | **507.1141**; |
| Taxifolin | 3.658 | C_15_ H_12_ O_7_ | 303.0506 | 304.0581 | 304.0583 | 93.66 | -0.69 | **303.0506**; 300.9987 |
| Flavan-3-oles |  |  |  |  |  |  |  |  |
| Catechin | 0.894 | C_15_ H_14_ O_6_ | 289.0712 | 290.0785 | 290.0790 | 98.52 | -1.97 | 289.0720; **175.0612**; 115.0401 |
| 3-methyl-catechin | 0.482 | C_16_ H_16_ O_6_ | 303.0887 | 304.0954 | 304.0947 | 74.49 | 2.17 | **303.0844**; |
| Gallocatechin | 0.517 | C_15_ H_14_ O_7_ | 305.0671 | 306.0751 | 306.0740 | 72.84 | 3.77 | **305.0661**; |
| **Compound** | **R.T. (min)** | **Formula** | **Precursor [M-H]** | **Experimental Mass** | **Theorical Mass** | **Score** | **Difference (ppm)** | **Fragments** |
| Epicatechin | 1.636 | C_15_ H_14_ O_6_ | 289.0716 | 290.0789 | 290.0790 | 99.48 | -0.65 | **289.0714**; |
| Epicatechin-3-glucuronide | 2.950 | C_21_ H_22_ O_12_ | 465.1041 | 466.1101 | 466.1111 | 73.51 | -2.22 | **465.1018**; |
| Epicatechin gallate | 3.788 | C_22_ H_18_ O_10_ | 441.0826 | 442.0900 | 442.0900 | 98.98 | 0.04 | **441.0829**; |
| Epigallocatechin | 0.928 | C_15_ H_14_ O_7_ | 305.0655 | 306.0728 | 306.0740 | 90.36 | -3.81 | **305.0633**; |
| Procyanidin B1 | 0.591 | C_30_ H_26_ O_12_ | 577.1346 | 578.1419 | 578.1424 | 98.37 | -0.86 | **577.1343**; |
| Procyanidin B2 | 1.231 | C_30_ H_26_ O_12_ | 577.1347 | 578.1420 | 578.1424 | 98.35 | -0.77 | **577.1353**; |
| Procyanidin B3 | 3.013 | C_30_ H_26_ O_12_ | 577.1358 | 578.1434 | 578.1424 | 92.20 | 1.72 | **577.1305**; |
| Procyanidin B4 | 3.822 | C_30_ H_26_ O_12_ | 577.1344 | 578.1416 | 578.1424 | 74.48 | -1.48 | **577.1332**; |
| Procyanidin C1 | 0.861 | C_45_ H_38_ O_18_ | 865.1968 | 866.2041 | 866.2058 | 95.97 | -2.00 | **865.1969**; 289.0714; |
| Procyanidin C2 | 2.613 | C_45_ H_38_ O_18_ | 865.1968 | 866.2043 | 866.2058 | 96.26 | -1.72 | **865.1964**; 289.0805; 197.0507 |

Fragments in bold express the most abundant ion identified.
